# The association between nutritional status, sarcopenia, and depressive symptoms in elderly Chinese patients receiving maintenance hemodialysis: a hospital-based cross-sectional study

**DOI:** 10.3389/fnut.2026.1720460

**Published:** 2026-02-18

**Authors:** Lin Huang, Yan Zhang, Jinbao Wang, Jiajun Zhou

**Affiliations:** 1 Blood Purification Center, The First Affiliated Hospital of Wannan Medical College (Yijishan Hospital of Wannan Medical College), Wuhu, China; 2 Department of Nephrology, The Second Affiliated Hospital of Anhui Medical University, Hefei, China

**Keywords:** association, depressive symptoms, maintenance hemodialysis, nutritional status, sarcopenia

## Abstract

**Background:**

Depressive symptoms are common among elderly patients undergoing maintenance hemodialysis (MHD), and factors such as nutritional status and sarcopenia may contribute to their mental health deterioration. This study aimed to investigate the relationship between nutritional status, sarcopenia, and depressive symptoms in elderly Chinese MHD patients.

**Methods:**

A cross-sectional study was conducted involving 324 elderly Chinese patients undergoing MHD. Nutritional status was assessed using the Geriatric Nutritional Risk Index (GNRI), and all patients were categorized into four quartiles Q1-Q4. Sarcopenia was assessed using the SARC-F (Strength, Assistance with Walking, Rising from a Chair, Climbing Stairs, and Falls) scale. Depressive symptoms were evaluated using the Patient Health Questionnaire (PHQ-9). Spearman rank correlation analysis and binary logistic regression models were used to examine the association between nutritional status and sarcopenia with depressive symptoms, adjusting for various clinical and biochemical factors.

**Results:**

Among the 324 patients, 17.00% patients (*n* = 55) had depressive symptoms with 54.00% of males and a median age of 63.24 years, and 21.60% patients (*n* = 70) had sarcopenia. There was a significant decrease in the prevalence of depressive symptoms in patients with the increasing quartiles of GNRI values. The Spearman correlation and binary logistic regression analyses revealed the potential associations between depression and several factors, such as age, albumin, C-reactive protein, GNRI, and sarcopenia score (*p* < 0.05). The results suggested that lower GNRI values were significantly associated with increased odds of depressive symptoms, with patients in the lowest quartile (Q1) showing the highest odds (OR: 11.782, *p* < 0.001). Sarcopenia was also strongly linked to depressive symptoms, with patients with sarcopenia having significantly higher odds of depression (OR: 7.383, *p* < 0.001). These associations remained significant after adjusting for multiple factors, including age, sex, antecedents, BMI, and kidney function markers.

**Conclusion:**

Poor nutritional status and sarcopenia are independently and significantly associated with depressive symptoms in elderly Chinese MHD patients. Interventions aimed at improving nutritional status and addressing sarcopenia may be beneficial for enhancing mental health and quality of life in this population. Further longitudinal studies are needed to confirm these findings and explore potential therapeutic strategies.

## 1 Introduction

The aging population in China is growing rapidly, and with this demographic shift comes an increased prevalence of chronic conditions such as chronic kidney disease (CKD). End-stage renal disease (ESRD) is a significant health concern, as it often necessitates long-term treatments like blood purification (maintenance hemodialysis (MHD) and peritoneal dialysis) and kidney transplantation, which, although life-saving, introduces various complications that impact patients′ physical and psychological well-being (1, 2). MHD patients (classified as CKD Stage 5, end-stage renal failure) were defined as individuals receiving regular hemodialysis treatment for at least 3 months, with a frequency of 3 sessions per week (3). Among the most common and debilitating issues faced by elderly hemodialysis patients are musculoskeletal weakness, osteoporosis, sarcopenia (age-related muscle loss), cognitive impairment, and depressive symptoms (4–7). These factors, often intertwined, can severely affect the overall quality of life and exacerbate clinical outcomes in this patient group.

Nutritional status plays a pivotal role in the prognosis of patients undergoing hemodialysis. Malnutrition is common in this population, with up to 30–50% of hemodialysis patients experiencing some forms of malnutrition (8). This can result from multiple factors, including insufficient dietary intake, metabolic acidosis, systemic inflammation, bowel flora alteration, metabolic disturbances, and hormonal dysregulation, associated with CKD. Malnutrition in ESRD patients is associated with increased morbidity, mortality, and prolonged hospitalization (9). Monitoring biochemical markers, such as serum albumin and C-reactive protein, can help assess nutritional deficiencies and inflammatory states in these patients. Low albumin levels, in particular, have been linked to poor survival rates in ESRD patients, highlighting the need for appropriate nutritional management (10). Sarcopenia, the loss of skeletal muscle mass and strength due to aging, is another prevalent issue in elderly hemodialysis patients. It is often worsened by CKD-related factors such as inflammation, metabolic acidosis, and reduced physical activity due to frequent dialysis sessions (11). Sarcopenia in ESRD patients contributes to frailty, functional impairment, and a higher risk of falls and disability. It also leads to poorer clinical outcomes, including increased hospitalization and mortality (12). Early identification and intervention for sarcopenia are crucial to improving physical function and overall health in these patients. In addition, depressive symptoms are commonly observed in elderly patients with ESRD. The burden of chronic illness, along with the physical limitations of hemodialysis treatment, can lead to feelings of hopelessness, social isolation, and loss of self-worth (13, 14). Depression in hemodialysis patients is not only a quality-of-life issue but also negatively impacts treatment adherence and clinical outcomes. Studies have shown that depression can exacerbate other conditions, such as chronic illnesses or age-related physical decline, making it an important factor to address in the holistic care of elderly hemodialysis patients (15). As the elderly population in China continues to rise, understanding the interplay between nutrition, muscle health, and mental well-being becomes increasingly important. By 2030, it is estimated that more than 20% of China′s population will be over the age of 60, a demographic shift that will lead to a greater number of elderly individuals requiring ESRD management and dialysis. Thus, understanding the associations between nutritional status, sarcopenia, and depressive symptoms in elderly patients undergoing hemodialysis is essential for improving their health outcomes and quality of life.

Previous research has established a high prevalence of malnutrition, sarcopenia, and depressive symptoms individually in the hemodialysis population, and has begun to explore their pairwise relationships. Studies have reported associations between poor nutritional status (assessed by various tools) and increased risk of depression, as well as links between sarcopenia (or physical frailty) and poorer mental health outcomes in patients with end-stage renal disease (16–19). However, existing literature often examines these factors in isolation or within broader patient groups, with limited focus on the elderly demographic specifically undergoing MHD. Furthermore, there is a notable scarcity of studies that concurrently investigate the triad of nutrition, muscle health, and depression using tools specifically validated or recommended for geriatric and dialysis populations, such as the Geriatric Nutritional Risk Index (GNRI) for nutrition and the SARC-F questionnaire for sarcopenia screening. This lack of integrated analysis, particularly within the context of Chinese elderly MHD patients, leaves a gap in understanding whether these factors are independently or synergistically associated with depressive symptoms when considered together. Moreover, whether these associations are independent of key confounders remains unclear. Therefore, this study aims to fill this gap by investigating the interrelationships between nutritional status (using GNRI), sarcopenia risk (using SARC-F), and depressive symptoms in a well-defined cohort of elderly Chinese MHD patients. Therefore, based on the established links between nutritional decline, musculoskeletal deterioration, and psychological distress in chronic disease populations, this study aims to investigate their interrelationships in a specific clinical setting. We hypothesize that: (1) poorer nutritional status, as indicated by lower GNRI scores, is independently associated with a higher prevalence of depressive symptoms in elderly Chinese MHD patients; (2) the presence of sarcopenia, screened by the SARC-F scale, is independently associated with a higher likelihood of depressive symptoms; and (3) these associations remain statistically significant after adjusting for a comprehensive set of demographic, clinical, and biochemical confounding factors. By assessing these factors in tandem, we hope to gain insights into their interconnections and identify potential areas for intervention. The findings of this study may inform clinical practices and contribute to the development of targeted strategies to improve the health and well-being of elderly patients on hemodialysis.

## 2 Patients and methods

### 2.1 Study subjects

**Figure 1:**
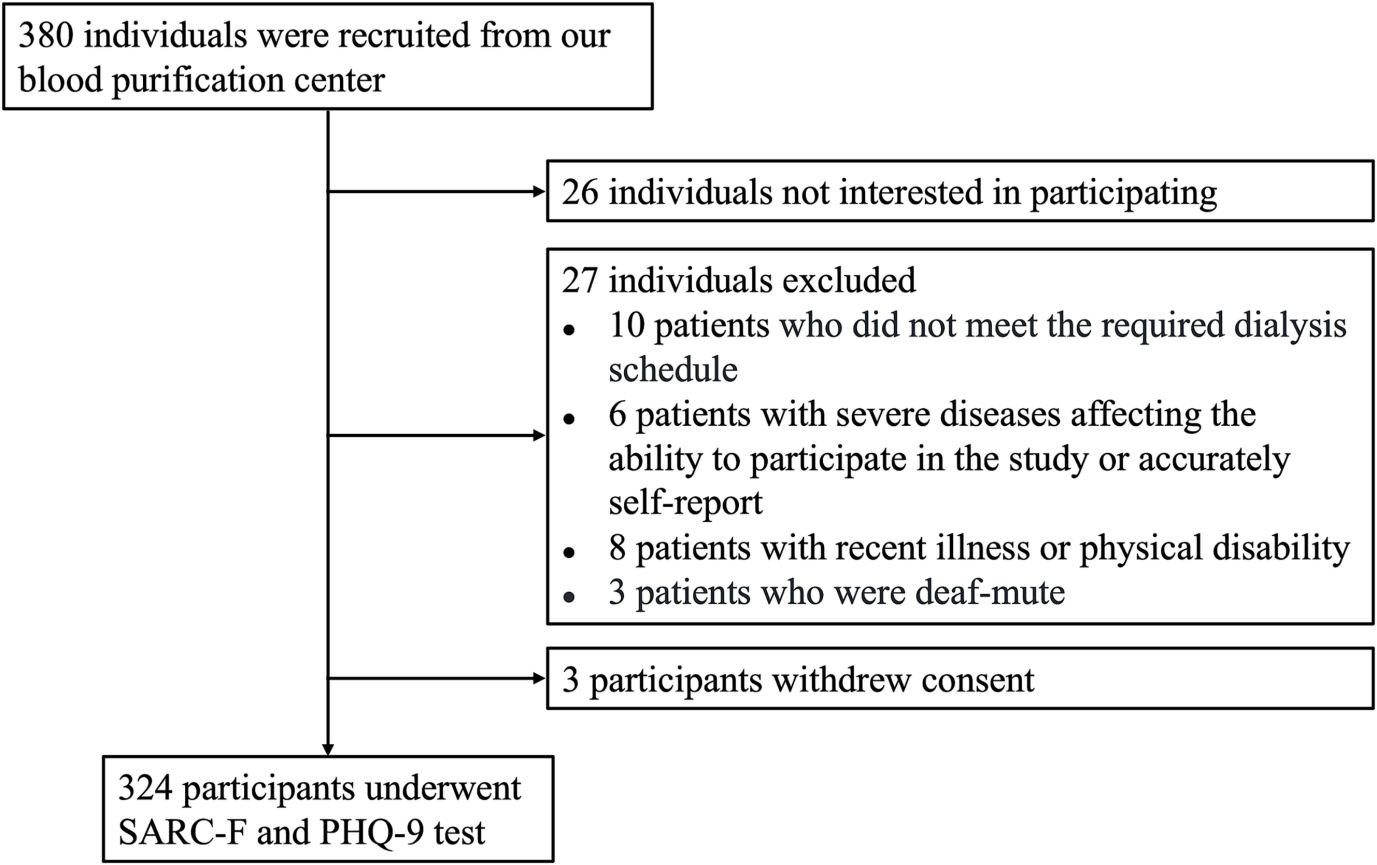
Participant screening flow diagram. A total of 380 individuals were assessed for eligibility. Of these, 26 chose not to participate, 27 were excluded based on the inclusion criteria, and 3 withdrew their consent. Consequently, 324 participants completed the SARC-F and PHQ-9 assessments.

A cross-sectional study was conducted at the Affiliated Yijishan Hospital of Wannan Medical College from February 2025 to July 2025. All elderly patients (≥ 60 years) undergoing MHD for over 6 months at the Blood Purification Center of our hospital constituted the source population. Consecutive enrollment was applied to all eligible patients during this period to minimize selection bias. Out of 380 initially screened patients, 56 were excluded according to the exclusion criteria, resulting in a final sample of 324 participants (see Figure 1 for the flow diagram). The inclusion criteria were as follows: (1) age ≥ 60 years; (2) undergoing MHD for a minimum of 3 months with a treatment duration of 4 h per session, three times per week; (3) clinically stable condition (no hospitalization required within the last 3 months). The exclusion criteria included: (1) irregular or inadequate dialysis treatment; (2) communication difficulties or refusal to complete the questionnaire; (3) malignant tumor diseases or severe psychiatric disorders affecting the ability to participate in the study or accurately self-report; (4) physical disability or other illnesses within the past 3 months. The study protocol received approval from the Ethics Committee of Wannan Medical College Affiliated Yijishan Hospital (Blood Purification Center, approval number: 20250218). All participants were informed of the study details and provided written informed consent before enrollment, in accordance with the Declaration of Helsinki.

### 2.2 Data collection

A structured face-to-face interview was conducted to gather demographic information (e.g., age, gender) and clinical characteristics (e.g., comorbidities like diabetes and hypertension, medication usage, and duration of hemodialysis). Clinical data were collected from all participants at a single time point, before their scheduled dialysis session. Blood samples were collected to analyze various biochemical parameters, including hemoglobin levels, 25-hydroxyvitamin D (25(OH)D), serum albumin concentration, and markers of renal function (such as blood urea nitrogen, serum creatinine, and uric acid). Lipid profile components [total cholesterol, triglycerides, low-density lipoprotein cholesterol (LDL-C)], and electrolytes (potassium, sodium, phosphorus, calcium, and magnesium) were also measured. Additionally, C-reactive protein, intact parathyroid hormone (iPTH) levels, and single-pool Kt/V (spKt/V) were simultaneously assessed. To ensure the accuracy and consistency of data collection, standardized measurement protocols and quality control procedures were implemented in this study. All blood samples were collected pre-dialysis and analyzed using standard laboratory methods: serum albumin was measured by the bromocresol green method, C-reactive protein was determined via immunoturbidimetric assay, and other biochemical parameters were analyzed using the hospital laboratory′s automated analyzers. For the questionnaire administration, all interviewers received uniform training on the standardized administration of the Patient Health Questionnaire (PHQ-9) and SARC-F scales. Interviews were conducted face-to-face in a quiet, private setting to ensure accurate responses and patient comfort. To address potential self-report bias, the PHQ-9 was completed via a combined method of patient self-assessment with interviewer clarification available when needed. Participants were assured of the confidentiality of their responses and that there were no right or wrong answers. All collected questionnaire data and laboratory results were independently entered into an electronic database by two research assistants, followed by cross-verification. Any discrepancies were resolved by referring back to the original source records, thereby ensuring data completeness and accuracy. All participants received regular hemodialysis treatments three times a week, with each session lasting 4 h. Hemodialysis was performed using a “Gambro” machine paired with “Gambro” Polyflux L capillary dialyzers. Among these patients, vascular access for hemodialysis was primarily through an autologous arteriovenous fistula, used by 95.3% of patients, while 2.8% had long-term central venous catheter access, and 1.9% used grafted vascular access. Bicarbonate-based dialysate was employed for all dialysis sessions.

### 2.3 Assessment of nutritional status

Nutritional status was assessed using both biochemical analyses and anthropometric measurements. Biochemical parameters were measured from blood samples as described above. Anthropometric data were obtained by calculating body mass index (BMI) using the Equation 1.


$$\mathrm{BMI}=\frac{\text{weight}}{\text{heigh}t^{2}}(\mathrm{kg}/m^{2})$$ (1)

The Geriatric Nutritional Risk Index (GNRI) was calculated to evaluate nutritional status based on the serum albumin level, height, body weight, and ideal body weight of patients. The ideal body weight for men and women were calculated using the following Lorentz Equations 2 and 3, respectively (20):


$$\text{For}\;\mathrm{men}:\text{Ideal body weight}=\text{height}-100-[\frac{\text{height}-150}{4}]$$ (2)

$$\text{For women}:\text{Ideal body weight}=\text{height}-100-[\frac{\text{height}-150}{2.5}]$$ (3)

The GNRI value was calculated through the Equation 4:


$$\text{GNRI}=[14.89\times \text{albumin}(g/\mathrm{dL})]+[41.7\times (\frac{\text{weight}}{\text{ideal body weight}})]$$ (4)

### 2.4 Assessment of sarcopenia

Sarcopenia was assessed using the SARC-F (Strength, Assistance with Walking, Rising from a Chair, Climbing Stairs, and Falls) scale, a tool developed by Malmstrom in 2013 and later adapted into Chinese (21, 22). This scale includes five items designed to evaluate various aspects of physical function and frailty, focusing on the difficulties individuals experience in performing common daily activities. The five items are as follows: (1) Carrying and Lifting 10 Pounds: How much difficulty do you have when carrying and lifting a 10-pound object? (None = 0, Some = 1, A lot or Unable = 2); (2) Walking Across a Room: How much difficulty do you have when walking across a room? (None = 0, Some = 1, A lot, Need Aids, or Unable = 2); (3) Transferring from a Bed or Chair: How much difficulty do you have when transferring from a bed or chair? (None = 0, Some = 1, A lot or Unable Without Help = 2); (3) Climbing Stairs: How much difficulty do you have when climbing about 10 stairs? (None = 0, Some = 1, A lot or Unable = 2); (4) Frequency of Falls: How many times have you fallen in the last 12 months? (None = 0, 1–3 Falls = 1, 4 or More Falls = 2). Each item is scored from 0 to 2, with higher scores indicating greater difficulty or more frequent falls. The total score ranges from 0 to 10, with a score of 4 or higher indicating the possible presence of sarcopenia and an increased risk of adverse outcomes. This scale has shown strong reliability and validity, particularly in elderly populations, and has been effectively used to identify individuals at risk for sarcopenia in Chinese cohorts (23, 24). The data gathered from these items allows for an objective assessment of an individual’s physical condition, particularly strength, balance, and coordination. Higher scores suggest greater difficulty in performing routine tasks, highlighting potential issues related to frailty and functional decline.

### 2.5 Assessment of depressive symptoms

The PHQ-9 is a widely recognized and validated tool for screening depression, used across various populations with chronic conditions, including those with renal disease. In particular, among patients with ESRD, research has shown that the PHQ-9 is an effective measure for identifying depressive symptoms, demonstrating a strong agreement with clinical diagnoses of depression (25–27). Moreover, the PHQ-9 has been found to correlate well with other depression assessment instruments, such as the Beck Depression Inventory (27). In this study, the PHQ-9 was employed to assess depression in hemodialysis patients. The questionnaire consists of nine self-reported items, addressing (1) loss of interest or pleasure, (2) feelings of sadness or hopelessness, (3) sleep disturbances, (4) fatigue or lack of energy, (5) appetite changes, (6) low self-esteem, (7) difficulty concentrating, (8) motor restlessness or slowing, and (9) thoughts of self-harm or suicide. This tool aligns with the diagnostic criteria for depression specified in the Diagnostic and Statistical Manual of Mental Disorders, Fourth Edition (DSM-IV) (25). Each item is rated on a 4-point scale (0 = not at all, 1 = several days, 2 = more than half the days, and 3 = nearly every day). The total score, ranging from 0 to 27, reflects the cumulative frequency of depressive symptoms experienced over the preceding 2 weeks. A score of ≥ 10 has been shown to be both valid and reliable for diagnosing depression in dialysis patients, as evidenced by prior studies (26, 27).

### 2.6 Variable definitions

For clarity in analysis and interpretation, all key study variables were explicitly defined as follows. The primary outcome variable was the presence of depressive symptoms, dichotomized using a PHQ-9 score of ≥10, a validated cutoff for screening depression in dialysis populations. The primary exposure variables were nutritional status and sarcopenia. Nutritional status was assessed using the GNRI. To examine the dose–response relationship, GNRI values were categorized into quartiles (Q1-Q4) based on the distribution within our study cohort. Sarcopenia was defined as a SARC-F scale score ≥ 4, in accordance with its established use as a screening tool. Potential confounding variables included demographic factors (age, sex), clinical characteristics (body mass index, history of diabetes and hypertension, dialysis vintage), and pertinent laboratory parameters (e.g., serum albumin, C-reactive protein, renal function markers). The selection of these covariates was based on their established or plausible biological and clinical relevance to the exposures and outcome, as supported by existing literature.

### 2.7 Statistical analysis

For quantitative data that follow a normal distribution and exhibit homogeneity of variance, the mean ± standard deviation (x̄ ± SD) was calculated. The independent samples t-test was used for comparing two groups, while one-way ANOVA was employed for comparing multiple groups. For non-normally distributed data, the median (*P_25_*, *P_75_*) was reported, with the Mann–Whitney U test for pairwise comparisons and the Kruskal-Wallis H test for comparisons across more than two groups. Categorical data were presented as frequency and percentage, with the Chi-square test or Fisher’s exact test used for group comparisons. The GNRI values of all patients were categorized into four groups based on quartiles, including Q1 (GNRI ≤ 97.39), Q2 (97.39 < GNRI ≤ 102.98), Q3 (102.98 < GNRI ≤ 107.91), and Q4 (GNRI > 107.91), to examine the dose–response relationship across the spectrum of nutritional status and to avoid reliance on a single, potentially arbitrary cutoff value in this specific population. To assess the relationship between PHQ-9 scores and various potential factors (demographic characteristics and biochemical measurements), the Spearman rank correlation test was applied. Univariate logistic regression was used to identify the potential factors influencing depressive symptoms in MHD patients. Furthermore, multivariate logistic regression models were employed to investigate the associations between nutritional status, sarcopenia, and depressive symptoms in this population. In the statistical analysis, to control for potential confounding, covariates for inclusion in the multivariate logistic regression models were selected based on a combination of univariate analysis (*p* < 0.05) and clinical relevance established from prior literature. These covariates included age, sex, history of diabetes, history of hypertension, BMI, serum albumin, C-reactive protein, and markers of kidney function. The rate of missing data for key variables in this study was less than 1%; therefore, a complete-case analysis approach was employed for handling missingness. A significance level of *p* < 0.05 was considered statistically significant. All statistical analyses were performed using SPSS software (version 27.0, IBM Corporation, Armonk, NY, USA).

## 3 Results

A total of 380 MHD patients were initially evaluated for eligibility to participate in the study (Figure 1). Among them, 27 individuals were excluded due to not meeting the inclusion criteria, including non-compliance with the prescribed dialysis regimen (*n* = 10), severe diseases affecting the ability to participate in the study or accurately self-report (*n* = 6), recent physical illness or disability (*n* = 8), and being deaf (*n* = 3). Additionally, 26 patients who met the criteria decided not to participate. As a result, 327 patients initially agreed to take part in the study, but three later withdrew their consent prior to depression testing. Ultimately, 324 participants completed the full SARC-F and PHQ-9 assessments. The determination of the sample size in this study was based on the available population of elderly MHD patients at our center during the predefined six-month recruitment period, which aligns with the cross-sectional design aiming to assess a representative snapshot of this clinical population. A post-hoc calculation was performed to address the statistical power. Given the final sample size of 324 participants, an observed odds ratio of 7.383 for the association between sarcopenia and depressive symptoms, an alpha level of 0.05, and accounting for up to 10 covariates in the multivariable logistic regression model, the achieved statistical power exceeded 90%. This confirms that the study size was adequate to detect significant associations with a high degree of reliability.

In this cross-sectional study, a total of 324 elderly Chinese patients undergoing MHD were included. The demographic information and clinical laboratory data of all participants were presented in Table 1. The cohort was divided into two groups based on the presence of depressive symptoms, with 269 patients classified as non-depressed and 55 as depressed, as determined by the PHQ-9 score. The percentages of patients with depressive symptoms and sarcopenia were 17.00% (*n* = 55) and 21.60% (*n* = 70), respectively. The mean age of the entire participants was 63.24 years (median value), with depressed patients being significantly older (70.25 years) compared to non-depressed patients (62.54 years) (*p* < 0.001). There were no significant gender and medical antecedent differences between the two groups (*p* > 0.05). In terms of clinical laboratory parameters, no significant differences were observed between the groups, such as hemoglobin, 25(OH)D, creatinine, blood urea nitrogen, uric acid, lipid profile components, and electrolyte levels. However, the depressed group had a significantly lower albumin concentration (42.91 g/L *vs.* 40.08 g/L, *p* = 0.046) compared to the non-depressed group. In addition, C-reactive protein levels were notably higher in the depressed group (4.56 mg/L *vs.* 2.94 mg/L, *p* = 0.041), with an interquartile range (IQR) of 7.35 mg/L for the depressed group and 4.84 mg/L for the non-depressed group. The analysis revealed a significant difference in GNRI values between the non-depressed and depressed groups. The mean GNRI for non-depressed patients was 104.15 ± 7.94, while depressed patients had a significantly lower GNRI of 96.27 ± 7.85 (*p* < 0.001). Sarcopenia, assessed by the SARC-F score, was more prevalent in depressed patients, with a median score of 4.88 (IQR = 4.07) compared to 2.45 (IQR = 1.63) in non-depressed patients (*p* < 0.001). Furthermore, 60.00% of depressed patients had sarcopenia, in contrast to only 13.80% in non-depressed patients (*p* < 0.001). These findings suggest a strong association between lower GNRI values and the presence of depressive symptoms in MHD patients, with sarcopenia further exacerbating this relationship.

**Table 1:** Demographic, clinical, and biochemical characteristics of elderly MHD patients with and without depressive symptoms.

Characteristics	Total, *n* = 324	Nondepressed, *n* = 269	Depressed, *n* = 55	*p* value
Age, years, (range)	63.24 (61.25, 68.29)	62.54 (61.03, 66.13)	70.25 (66.36, 74.90)	<0.001
Males, *N* (%)	175 (54.00%)	148 (55.00%)	27 (49.10%)	0.422
Females, *N* (%)	149 (46.00%)	121 (45.00%)	28 (50.90%)	0.422
Antecedents, *N* (%)				
Hypertension	114 (35.20%)	97 (36.10%)	17 (30.90%)	0.466
Diabetes	42 (13.00%)	37 (13.80%)	5 (9.10%)	0.348
Dyslipidemia	16 (4.90%)	12 (4.50%)	4 (7.30%)	0.381
Duration of hemodialysis (months)	84.00 (43.38, 135.50)	80.00 (41.38, 135.70)	94.00 (50.67, 133.00)	0.256
BMI, kg/m^2^	21.65 (19.27, 24.07)	21.79 (19.59, 24.13)	19.90 (17.56, 23.43)	0.006
GNRI	102.81 ± 8.45	104.15 ± 7.94	96.27 ± 7.85	<0.001
Sarcopenia score	2.68 (1.82, 3.92)	2.45 (1.66, 3.29)	4.88 (2.64, 6.71)	<0.001
Sarcopenia, *N* (%)	70 (21.60%)	37 (13.80%)	33 (60.00%)	<0.001
PHQ-9 score	6.86 (4.43, 9.00)	6.07 (3.95, 7.71)	11.25 (10.20, 12.45)	<0.001
Depressive symptoms, *N* (%)	55 (17.00%)	0 (0%)	55 (100%)	-
Clinical laboratory characteristics				
Hemoglobin, g/L	113.23 ± 16.64	113.58 ± 16.36	111.51 ± 18.02	0.402
Creatinine, μmol/L	766.75 (440.00, 1016.80)	784.70 (424.38, 1038.18)	639.10 (452.30, 943.10)	0.272
Blood urea nitrogen, mmol/L	19.39 (10.43, 26.13)	20.27 (10.61, 26.51)	16.46 (9.68, 23.93)	0.169
Uric acid, μmol/L	408.90 ± 124.82	410.82 ± 126.38	399.47 ± 117.57	0.540
Potassium, mmol/L	4.73 (4.02, 5.30)	4.74 (4.09, 5.27)	4.68 (3.60, 5.37)	0.632
Sodium, mmol/L	137.95 ± 2.96	138.05 ± 2.95	137.49 ± 3.01	0.201
Phosphorus, mmol/L	1.73 (1.42, 2.15)	1.71 (1.40, 2.16)	1.75 (1.52, 2.12)	0.799
Calcium, mmol/L	2.29 (2.15, 2.43)	2.30 (2.16, 2.44)	2.27 (2.14, 2.43)	0.714
Magnesium, mmol/L	1.05 (0.96, 1.13)	1.04 (0.97, 1.14)	1.06 (0.93, 1.12)	0.607
Albumin, g/L	41.43 (39.03, 43.30)	42.91 (40.09, 44.39)	40.08 (38.45, 42.63)	0.046
β2-Microglobulin, mg/L	36.35 (30.70, 44.78)	36.30 (30.55, 44.82)	36.50 (31.08, 43.33)	0.958
Iron, μmol/L	12.70 (9.45, 17.80)	12.83 (9.54, 18.53)	11.90 (9.28, 16.92)	0.221
Ferritin, μg/L	275.00 (55.30, 1640.00)	333.00 (58.50, 1742.50)	198.00 (40.85, 1077.50)	0.087
Transferrin, g/L	1.54 (1.19, 1.93)	1.55 (1.20, 1.91)	1.49 (1.17, 2.03)	0.876
C-Reactive protein, mg/L	3.06 (1.36, 6.91)	2.94 (1.31, 6.15)	4.56 (1.69, 9.04)	0.041
iPTH, pg./mL	192.95 (97.60, 359.73)	181.40 (97.08, 349.30)	229.90 (121.33, 408.25)	0.193
Triglyceride, mmol/L	1.43 (1.05, 2.20)	1.46 (1.07, 2.21)	1.41 (1.02, 2.15)	0.769
Total cholesterol, mmol/L	3.95 ± 1.10	3.95 ± 1.09	3.99 ± 1.14	0.780
LDL-C, mmol/L	2.30 (1.74, 2.78)	2.27 (1.73, 2.72)	2.35 (1.86, 2.83)	0.634
25(OH)D, ng/mL	49.50 (36.85, 66.88)	49.80 (36.79, 68.00)	47.58 (38.88, 61.80)	0.518
spKt/V	1.21 (1.19, 1.39)	1.23 (1.19, 1.40)	1.21 (1.17, 1.33)	0.083

Quantitative data that are normally distributed with equal variance are presented as mean ± standard deviation (x̄ ± SD). For data that do not follow a normal distribution, the median with interquartile range (*P*_25_, *P*_75_) is reported. Categorical variables are expressed as counts and percentages.

BMI, body mass index; 25(OH)D, 25-hydroxyvitamin D; LDL-C, low-density lipoprotein cholesterol; iPTH, intact parathyroid hormone; spKt/V, single-pool Kt/V.

In this study, we compared the clinical and laboratory characteristics of MHD patients with and without sarcopenia (Table 2). The sarcopenia group (*n* = 70) was significantly older (68.80 years *vs.* 62.71 years) compared to the non-sarcopenia group (*n* = 254) (*p* < 0.001). There were no significant differences between the two groups regarding gender distribution, the presence of hypertension, diabetes, dyslipidemia, or the duration of hemodialysis. However, depressive symptoms, as assessed by the PHQ-9 score, were markedly higher in the sarcopenia group (8.94, IQR = 4.37) compared to the non-sarcopenia group (6.13, IQR = 4.12) (*p* < 0.001). In terms of laboratory characteristics, the two groups did not show significant differences in hemoglobin, creatinine, blood urea nitrogen, uric acid, potassium, sodium, phosphorus, calcium, magnesium, albumin, iron, ferritin, transferrin, C-reactive protein, iPTH, triglycerides, total cholesterol, LDL-C, 25(OH)D, and spKt/V (*p* > 0.05 for all). The GNRI, a marker of nutritional status, was significantly lower in the sarcopenia group (95.47 ± 7.48) compared to the non-sarcopenia group (104.74 ± 7.55) (*p* < 0.001). Moreover, the sarcopenia group had significantly higher sarcopenia scores (6.16, IQR = 2.11) than the non-sarcopenia group (2.26, IQR = 1.32) (*p* < 0.001).

**Table 2:** Demographic, clinical, and biochemical characteristics of elderly MHD patients with and without sarcopenia.

Characteristics	Non-Sarcopenia, *n* = 254	Sarcopenia, *n* = 70	*p* value
Age, years, (range)	62.71 (61.12, 66.44)	68.80 (62.00, 73.56)	<0.001
Males, *N* (%)	139 (54.70%)	36 (51.40%)	0.624
Females, *N* (%)	115 (45.30%)	34 (48.60%)	0.624
Antecedents, *N* (%)			
Hypertension	93 (36.60%)	21 (30.00%)	0.305
Diabetes	34 (13.40%)	8 (11.40%)	0.666
Dyslipidemia	12 (4.70%)	4 (5.70%)	0.735
Duration of hemodialysis (months)	82.50 (41.50, 136.33)	85.67 (48.25, 132.00)	0.256
BMI, kg/m^2^	22.16 (20.00, 24.34)	18.20 (16.98, 20.80)	0.006
GNRI	104.74 ± 7.55	95.47 ± 7.48	<0.001
Sarcopenia score	2.26 (1.54, 2.86)	6.16 (5.00, 7.11)	<0.001
Sarcopenia, *N* (%)	0 (0%)	70 (100%)	-
PHQ-9 score	6.13 (3.90, 8.02)	8.94 (6.94, 11.31)	<0.001
Depressive symptoms, *N* (%)	22 (8.70%)	33 (47.10%)	<0.001
Clinical laboratory characteristics			
Hemoglobin, g/L	113.35 ± 16.87	112.77 ± 15.88	0.706
Creatinine, μmol/L	767.20 (442.60, 1056.70)	764.50 (433.10, 978.40)	0.272
Blood urea nitrogen, mmol/L	19.00 (10.30, 26.08)	19.80 (11.10, 26.86)	0.169
Uric acid, μmol/L	410.07 ± 125.83	404.62 ± 121.90	0.844
Potassium, mmol/L	4.73 (4.02, 5.27)	4.75 (4.04, 5.43)	0.632
Sodium, mmol/L	138.06 ± 2.90	137.57 ± 3.17	0.998
Phosphorus, mmol/L	1.72 (1.41, 2.17)	1.75 (1.47, 2.07)	0.799
Calcium, mmol/L	2.30 (2.16, 2.44)	2.28 (2.13, 2.42)	0.714
Magnesium, mmol/L	1.04 (0.97, 1.14)	1.05 (0.94, 1.12)	0.607
Albumin, g/L	41.68 (39.05, 43.34)	40.90 (38.72, 43.00)	0.210
β2-Microglobulin, mg/L	36.70 (30.60, 44.80)	35.10 (31.30, 43.50)	0.958
Iron, μmol/L	12.55 (9.51, 18.60)	13.77 (9.10, 17.27)	0.221
Ferritin, μg/L	312.50 (55.90, 1680.00)	223.00 (54.70, 1500.00)	0.087
Transferrin, g/L	1.57 (1.20, 1.92)	1.46 (1.19, 1.96)	0.876
C-Reactive protein, mg/L	3.15 (1.32, 6.57)	2.78 (1.41, 7.96)	0.106
iPTH, pg./mL	184.20 (103.40, 351.10)	212.70 (85.50, 383.90)	0.193
Triglyceride, mmol/L	1.47 (1.08, 2.28)	1.38 (1.02, 1.79)	0.769
Total cholesterol, mmol/L	3.93 ± 1.10	4.05 ± 1.10	0.289
LDL-C, mmol/L	2.32 (1.75, 2.72)	2.18 (1.70, 2.85)	0.634
25(OH)D, ng/mL	49.44 (36.70, 67.44)	49.57 (40.02, 63.54)	0.518
spKt/V	1.21 (1.19, 1.38)	1.21 (1.19, 1.40)	0.083

Quantitative data that are normally distributed with equal variance are presented as mean ± standard deviation (x̄ ± SD). For data that do not follow a normal distribution, the median with interquartile range (*P*_25_, *P*_75_) is reported. Categorical variables are expressed as counts and percentages.

BMI, body mass index; 25(OH)D, 25-hydroxyvitamin D; LDL-C, low-density lipoprotein cholesterol; iPTH, intact parathyroid hormone; spKt/V, single-pool Kt/V.

The clinical and biochemical characteristics of the study participants across the four GNRI quartiles (Q1 to Q4) are summarized in Table 3. The patients in the lower GNRI quartile (Q1) had a significantly longer duration of hemodialysis (101.67 months, median value) compared to those in higher quartiles (Q2: 88.00 months, Q3: 75.50 months, Q4: 68.00 months, median values), with a *p* value of 0.045. The PHQ-9 score, indicating the severity of depressive symptoms, was highest in Q1 (7.65) and progressively decreased across quartiles, with Q4 having the lowest score (4.89). A significant difference in depressive symptoms was observed across the quartiles (*p* < 0.001), with 35.80% of patients in Q1 reporting depressive symptoms, compared to 4.90% in Q4. Regarding the clinical laboratory parameters, there were no significant differences in creatinine, blood urea nitrogen, uric acid, potassium, sodium, phosphorus, calcium, magnesium, and other biochemical markers between the groups. However, albumin levels significantly increased across quartiles, with Q4 having the highest albumin level (42.07 g/L) compared to Q1 (40.70 g/L, *p* < 0.001). Iron levels also showed a significant upward trend, with the highest value in Q4 (13.88 μmol/L, *p* = 0.024). Furthermore, C-reactive protein levels showed a significant variation across the quartiles of GNRI values (*p* = 0.035). The levels were highest in the first quartile (Q1: 3.56 mg/L, IQR = 1.90–7.78), and decreased progressively in subsequent quartiles: Q2 (3.68 mg/L, IQR = 2.00–7.77), Q3 (2.47 mg/L, IQR = 1.09–5.32), and Q4 (2.92 mg/L, IQR = 1.00–7.98). This trend suggests a decrease in C-reactive protein levels as GNRI values increased. The body composition parameter BMI differed significantly across the quartiles. Patients in Q1 had the lowest BMI (19.80 kg/m^2^) compared to Q4 (22.63 kg/m^2^, *p* = 0.002). Sarcopenia scores also decreased across the quartiles, with Q1 having the highest median score (4.25), and Q4 the lowest (2.25, *p* < 0.001). The percentage of patients diagnosed with sarcopenia significantly decreased from 54.30% in Q1 to 6.20% in Q4 (*p* < 0.001).

**Table 3:** Clinical, biochemical characteristics, and PHQ-9 scores across different GNRI quartiles in elderly MHD patients.

Variable	Q1 (*n* = 81)	Q2 (*n* = 82)	Q3 (*n* = 80)	Q4 (*n* = 81)	*p* value
Age, years, (range)	64.33 (61.31, 70.36)	63.78 (61.46, 68.33)	62.95 (61.36, 71.50)	62.53 (60.86, 65.41)	0.072
Males, *N* (%)	35 (43.20%)	45 (54.90%)	49 (61.30%)	46 (56.80%)	0.123
Females, *N* (%)	46 (56.80%)	37 (45.10%)	31 (38.70%)	35 (43.20%)	0.123
Antecedents, *N* (%)					
Hypertension	29 (35.80%)	23 (28.00%)	29 (36.30%)	33 (40.70%)	0.395
Diabetes	11 (13.60%)	7 (8.50%)	8 (10.00%)	16 (19.80%)	0.146
Dyslipidemia	6 (7.40%)	3 (3.70%)	2 (2.50%)	5 (6.20%)	0.455
Duration of hemodialysis (months)	101.67 (51.00, 154.75)	88.00 (46.00, 146.00)	75.50 (40.00, 125.67)	68.00 (38.88, 125.00)	0.045
BMI, kg/m^2^	19.80 (17.58, 22.33)	22.03 (19.20, 24.23)	21.70 (20.18, 24.05)	22.63 (20.15, 24.65)	0.002
GNRI	92.33 ± 4.25	100.22 ± 1.68	105.18 ± 1.35	113.57 ± 4.95	<0.001
Sarcopenia score	4.25 (2.63, 6.50)	2.54 (1.73, 3.52)	2.30 (1.50, 3.00)	2.25 (1.54, 2.88)	<0.001
Sarcopenia, *N* (%)	44 (54.30%)	15 (18.30%)	6 (7.50%)	5 (6.20%)	<0.001
PHQ-9 score	7.65 (5.66, 10.93)	7.54 (5.18, 9.32)	6.60 (4.56, 8.22)	4.89 (3.34, 6.97)	<0.001
Depressive symptoms, *N* (%)	29 (35.80%)	17 (20.70%)	5 (6.30%)	4 (4.90%)	<0.001
Clinical laboratory characteristics					
Hemoglobin, g/L	111.80 ± 16.07	113.33 ± 17.20	114.90 ± 13.96	112.89 ± 19.06	0.698
Creatinine, μmol/L	735.80 (447.85, 926.00)	724.95 (386.80, 970.70)	796.05 (418.40, 1113.20)	803.40 (512.85, 1096.05)	0.095
Blood urea nitrogen, mmol/L	17.88 (10.74, 24.66)	18.94 (9.20, 26.44)	20.47 (10.25, 26.78)	21.14 (10.89, 25.97)	0.746
Uric acid, μmol/L	390.12 ± 121.32	406.39 ± 110.81	414.07 ± 134.10	425.10 ± 131.69	0.339
Potassium, mmol/L	4.57 (4.06, 5.33)	4.65 (3.85, 5.33)	4.80 (4.09, 5.29)	4.84 (3.99, 5.27)	0.874
Sodium, mmol/L	137.49 ± 3.06	138.24 ± 2.72	138.11 ± 3.21	137.98 ± 2.83	0.399
Phosphorus, mmol/L	1.76 (1.49, 2.16)	1.66 (1.35, 2.04)	1.70 (1.39, 2.12)	1.90 (1.44, 2.32)	0.178
Calcium, mmol/L	2.28 (2.13, 2.42)	2.27 (2.16, 2.42)	2.32 (2.14, 2.45)	2.31 (2.17, 2.44)	0.870
Magnesium, mmol/L	1.01 (0.93, 1.12)	1.05 (0.95, 1.14)	1.07 (0.99, 1.14)	1.05 (0.99, 1.14)	0.206
Albumin, g/L	40.70 (38.38, 42.37)	40.80 (38.43, 42.97)	42.55 (40.20, 43.94)	42.07 (39.55, 43.58)	<0.001
β2-Microglobulin, mg/L	35.50 (31.31, 42.72)	38.20 (31.10, 48.00)	33.70 (29.10, 43.63)	37.00 (31.95, 43.25)	0.242
Iron, μmol/L	10.93 (8.88, 16.32)	12.15 (9.88, 17.10)	12.90 (9.53, 19.80)	13.88 (10.63, 19.83)	0.024
Ferritin, μg/L	244.25 (87.28, 676.50)	171.00 (31.30, 2500.00)	339.50 (57.90, 1455.00)	684.00 (125.00, 2655.00)	0.054
Transferrin, g/L	1.49 (1.19, 1.89)	1.58 (1.23, 1.92)	1.61 (1.16, 2.09)	1.54 (1.21, 1.87)	0.806
C-Reactive protein, mg/L	3.56 (1.90, 7.78)	3.68 (2.00, 7.77)	2.47 (1.09, 5.32)	2.92 (1.00, 7.98)	0.035
iPTH, pg./mL	204.80 (101.48, 362.00)	184.60 (108.60, 362.80)	176.45 (112.60, 334.90)	202.90 (93.00, 450.43)	0.978
Triglyceride, mmol/L	1.28 (1.02, 1.79)	1.49 (0.99, 2.28)	1.53 (1.10, 2.28)	1.49 (1.14, 2.47)	0.130
Total cholesterol, mmol/L	4.09 ± 1.09	3.84 ± 1.10	3.87 ± 1.13	4.03 ± 1.08	0.454
LDL-C, mmol/L	2.35 (1.87, 2.93)	2.22 (1.67, 2.60)	2.21 (1.67, 2.62)	2.29 (1.91, 2.90)	0.249
25(OH)D, ng/mL	49.87 (41.24, 66.47)	47.39 (35.95, 64.27)	51.41 (35.71, 71.90)	47.47 (37.56, 64.57)	0.491
spKt/V	1.23 (1.19, 1.49)	1.21 (1.17, 1.37)	1.22 (1.17, 1.34)	1.24 (1.20, 1.40)	0.245

Quantitative data that are normally distributed with equal variance are presented as mean ± standard deviation (x̄ ± SD). For data that do not follow a normal distribution, the median with interquartile range (*P*_25_, *P*_75_) is reported. Categorical variables are expressed as counts and percentages.

BMI, body mass index; 25(OH)D, 25-hydroxyvitamin D; LDL-C, low-density lipoprotein cholesterol; iPTH, intact parathyroid hormone; spKt/V, single-pool Kt/V.

The associations between PHQ-9 score and others parameters were analyzed using Spearman rank correlation test (Table 4). We found significant associations between the GNRI quartiles and various clinical parameters. Patients in the lower GNRI quartile (Q1) exhibited a higher prevalence of depressive symptoms as indicated by the PHQ-9 score (*ρ* = −0.352, *p* < 0.001). In contrast, higher GNRI quartiles were associated with lower depressive symptoms. The correlation analysis of PHQ-9 scores revealed that depressive symptoms were significantly and positively correlated with several clinical parameters, including age (*ρ* = 0.302, *p* < 0.001) and sarcopenia score (*ρ* = 0.311, *p* < 0.001). BMI was significantly correlated with depressive symptoms (*ρ* = −0.156, *p* = 0.005). In addition, no significant correlations were found with laboratory parameters such as creatinine, hemoglobin, or albumin levels, except spKt/V (*ρ* = −0.130, *p* = 0.019).

**Table 4:** Spearman rank correlation analysis between PHQ-9 scores with clinical, demographic, and biochemical parameters.

Characteristics	PHQ-9 score	
	*ρ*	*p*
Age (years)	0.302	<0.001
Sex, *N* (%)	−0.049	0.380
Antecedents, *N* (%)		
Hypertension	−0.030	0.595
Diabetes	−0.036	0.520
Dyslipidemia	−0.011	0.848
Duration of hemodialysis (months)	0.108	0.052
BMI, kg/m^2^	−0.156	0.005
GNRI	−0.352	<0.001
Sarcopenia score	0.311	<0.001
Clinical laboratory characteristics		
Hemoglobin, g/L	−0.006	0.921
Creatinine, μmol/L	−0.033	0.554
Blood urea nitrogen, mmol/L	−0.023	0.686
Uric acid, μmol/L	−0.032	0.564
Potassium, mmol/L	−0.001	0.981
Sodium, mmol/L	−0.029	0.600
Phosphorus, mmol/L	−0.101	0.071
Calcium, mmol/L	−0.082	0.141
Magnesium, mmol/L	−0.091	0.102
Albumin, g/L	0.019	0.733
β2-Microglobulin, mg/L	−0.004	0.941
Iron, μmol/L	−0.042	0.452
Ferritin, μg/L	−0.108	0.051
Transferrin, g/L	−0.005	0.931
C-Reactive protein, mg/L	−0.007	0.903
iPTH, pg./mL	0.036	0.514
Triglyceride, mmol/L	0.016	0.777
Total cholesterol, mmol/L	−0.020	0.715
LDL-C, mmol/L	−0.035	0.528
25(OH)D, ng/mL	−0.057	0.307
spKt/V	−0.130	0.019

BMI, body mass index; 25(OH)D, 25-hydroxyvitamin D; LDL-C, low-density lipoprotein cholesterol; iPTH, intact parathyroid hormone; spKt/V, single-pool Kt/V.

The logistic regression analysis was conducted to further investigate the potential association between nutritional status, sarcopenia, and depressive symptoms in MHD patients (Table 5). The results indicated a significant association between several clinical and biochemical parameters and depressive symptoms in this patient cohort. Univariate analysis revealed that lower GNRI values were significantly associated with depressive symptoms (OR: 0.874, 95% CI: 0.835–0.914, *p* < 0.001), and this association remained significant in the multivariate analysis (OR: 0.885, 95% CI: 0.826–0.947, *p* < 0.001). Furthermore, the sarcopenia score was found to be a significant predictor of depressive symptoms, with both univariate (OR: 1.687, 95% CI: 1.446–1.969, *p* < 0.001) and multivariate analysis (OR: 1.353, 95% CI: 1.066–1.719, *p* = 0.013) indicating a strong positive correlation with depression severity. The clinical variables, including age and albumin levels, showed significant associations (*p* < 0.05) with depression in the univariate analysis, with age continuing to show significance in the multivariate model. However, other clinical and biochemical factors, including gender, hypertension, diabetes, and various laboratory parameters such as hemoglobin, uric acid, potassium, and phosphorus, were not found to have a significant impact on depressive symptoms after adjusting for other variables. Furthermore, to assess the robustness of our findings, sensitivity analyses were performed. These included: (1) using the commonly applied clinical cutoff for GNRI (< 92 *vs.* ≥ 92) instead of the quartile-based categorization, and (2) redefining depressive symptoms using an alternative PHQ-9 cutoff score (≥ 8). The results of these sensitivity analyses were largely consistent with the findings from the primary analysis, supporting the reliability of our main conclusions.

**Table 5:** Logistic regression analysis of clinical and biochemical parameters associated with depressive symptoms in elderly MHD patients.

Variable	Univariate analysis		Multivariate analysis	
	OR (95% *CI*)	*p* value	OR (95% *CI*)	*p* value
Age (years)	1.145 (1.094–1.198)	<0.001	1.167 (1.094–1.245)	<0.001
Females, *N* (%)	Ref.	-		
Males, *N* (%)	0.788 (0.441–1.409)	0.422		
Antecedents, *N* (%)				
Hypertension	0.793 (0.425–1.480)	0.467		
Diabetes	0.627 (0.235–1.675)	0.352		
Dyslipidemia	1.680 (0.521–5.416)	0.385		
Duration of hemodialysis (months)	1.003 (0.999–1.007)	0.158		
BMI, kg/m^2^	0.875 (0.800–0.958)	0.004		
GNRI	0.874 (0.835–0.914)	<0.001	0.885 (0.826–0.947)	<0.001
Sarcopenia score	1.687 (1.446–1.969)	<0.001	1.353 (1.066–1.719)	0.013
Clinical laboratory characteristics				
Hemoglobin, g/L	0.993 (0.975–1.010)	0.401		
Creatinine, μmol/L	1.000 (0.999–1.000)	0.324		
Blood urea nitrogen, mmol/L	0.980 (0.951–1.010)	0.191		
Uric acid, μmol/L	0.999 (0.997–1.002)	0.539		
Potassium, mmol/L	0.892 (0.659–1.207)	0.459		
Sodium, mmol/L	0.938 (0.851–1.035)	0.201		
Phosphorus, mmol/L	0.801 (0.461–1.393)	0.432		
Calcium, mmol/L	1.008 (0.337–3.016)	0.989		
Magnesium, mmol/L	0.611 (0.070–5.300)	0.655		
Albumin, g/L	0.945 (0.879–1.015)	0.045		
β2-Microglobulin, mg/L	0.994 (0.971–1.016)	0.578		
Iron, μmol/L	0.966 (0.924–1.010)	0.132		
Ferritin, μg/L	1.000 (1.000–1.000)	0.088		
Transferrin, g/L	1.043 (0.649–1.674)	0.862		
C-Reactive protein, mg/L	1.000 (0.982–1.018)	0.972		
iPTH, pg./mL	1.000 (0.999–1.001)	0.928		
Triglyceride, mmol/L	0.922 (0.716–1.188)	0.532		
Total cholesterol, mmol/L	1.038 (0.798–1.352)	0.708		
LDL-C, mmol/L	1.158 (0.802–1.672)	0.434		
25(OH)D, ng/mL	0.997 (0.985–1.008)	0.580		
spKt/V	0.731 (0.308–1.736)	0.477		

BMI, body mass index; 25(OH)D, 25-hydroxyvitamin D; LDL-C, low-density lipoprotein cholesterol; iPTH, intact parathyroid hormone; spKt/V, single-pool Kt/V.

The associations between nutritional status, sarcopenia, and depressive symptoms in elderly MHD patients were further analyzed across four models using the Q4 (normal GNRI value) and non-sarcopenia groups as references. Table 6 presented the odds ratios (ORs) for depressive symptoms in relation to GNRI quartiles and sarcopenia status. The unadjusted analysis (Model 1) revealed a strong association between the lower GNRI quartiles (Q1 and Q2) and increased odds of depressive symptoms. Patients in Q1 (< 97.39) had a significantly higher likelihood of depression, with an OR of 10.736 (95% CI: 3.563–32.348, *p* < 0.001). Similarly, Q2 (97.39–102.98) patients had a notable increased risk of depressive symptoms (OR: 5.035, 95% CI: 1.613–15.713, *p* = 0.005), while Q3 showed no significant association with depression (*p* = 0.718). The association between lower GNRI values and depressive symptoms remained significant in the adjusted models (Models 2, 3, and 4), with the odds increasing as the GNRI values decreased. Notably, patients in Q1 had the highest OR in Model 4 (OR: 11.728, 95% CI: 3.383–41.034, *p* < 0.001), suggesting a robust link between poor nutritional status and depression. Additionally, sarcopenia was strongly associated with depressive symptoms across all models. Patients with sarcopenia had significantly higher odds of depression compared to those without sarcopenia. The unadjusted OR for sarcopenia was 9.405 (95% CI: 4.952–17.862, *p* < 0.001). This association remained significant after adjusting for sex, age, and duration of hemodialysis (Model 2: OR: 7.006, 95% CI: 3.525–13.926, *p* < 0.001), and further strengthened in Models 3 and 4 (Model 4: OR: 7.383, 95% CI: 3.631–15.011, *p* < 0.001). This indicates that sarcopenia is a significant and independent predictor of depressive symptoms in elderly MHD patients.

**Table 6:** Association of nutritional status (GNRI) and sarcopenia with depressive symptoms in elderly MHD patients.

Group	Model 1		Model 2		Model 3		Model 4	
	OR (95% *CI*)	*p* value	OR (95% *CI*)	*p* value	OR (95% *CI*)	*p* value	OR (95% *CI*)	*p* value
Q1: < 97.39	10.736 (3.563–32.348)	<0.001	10.302 (3.099–34.240)	<0.001	10.357 (3.106–34.540)	<0.001	11.782 (3.383–41.034)	<0.001
Q2: 97.39–102.98	5.035 (1.613–15.713)	0.005	3.982 (1.151–13.770)	0.029	4.058 (1.162–14.175)	0.028	4.303 (1.195–15.491)	0.026
Q3: 102.98–107.91	1.283 (0.332–4.964)	0.718	0.819 (0.191–3.501)	0.787	0.839 (0.195–3.611)	0.814	0.906 (0.208–3.949)	0.895
Q4: > 107.91	1.00	-	1.00	-	1.00	-	1.00	-
Sarcopenia (yes)	9.405 (4.952–17.862)	<0.001	7.006 (3.525–13.926)	<0.001	6.995 (3.514–13.927)	<0.001	7.383 (3.631–15.011)	<0.001
Sarcopenia (no)	1.00	-	1.00	-	1.00	-	1.00	-

Model 1: Unadjusted model.

Model 2: Adjusted sex, age, and duration of HD based on Model 1.

Model 3: Adjusted hypertension, diabetes, and dyslipidemia based on Model 2.

Model 4: Adjusted BMI, hemoglobin, creatinine, blood urea nitrogen, uric acid, and albumin based on Model 3.

## 4 Discussion

This cross-sectional study explored the complex relationships among nutritional status, sarcopenia, depressive symptoms, and renal function in elderly MHD patients. These findings emphasize the critical role of malnutrition and muscle loss in the development of depression and suggest that early interventions targeting these factors can significantly improve both physical and mental health in this vulnerable population (28).

Depressive symptoms are commonly observed in patients receiving hemodialysis, representing a significant psychological challenge. These symptoms are often associated with various adverse clinical outcomes, including poor nutritional status, reduced treatment adherence, impaired cognitive function, lower quality of life, and increased mortality (29). In our study, 17.00% of MHD patients exhibited depressive symptoms, as indicated by a PHQ-9 score ≥ 10, which was consistent with the findings from previous research. The prevalence of depression in dialysis patients has been reported to range from 20 to 30% (30). For example, Mapes et al. (2003) and Li et al. (2020) found depression rates of 20 and 15.2%, respectively (31, 32). Similarly, a study examining elderly CKD patients noted a 23% prevalence using the 15-item Geriatric Depression Scale (GDS-15) (33). Variability in depression prevalence across studies can be attributed to factors such as sample size, geographic location, dialysis method, depression screening tools, medication usage, comorbidities, and access to healthcare services.

Currently, there is limited research examining the association between nutritional status, sarcopenia, and depressive symptoms in elderly Chinese patients undergoing hemodialysis. This study found that patients with depression had significantly lower GNRI values alongside higher PHQ-9 and sarcopenia scores. These results are consistent with previous studies that have linked reduced GNRI value to malnutrition, sarcopenia, muscle loss, and an increased risk of mortality in CKD populations (34). A low GNRI reflects poor nutritional status, which emerges as an independent risk factor for depression in dialysis patients (35). Further analysis revealed that patients with depression generally had lower GNRI values, underscoring the pivotal role of malnutrition in the onset of depressive symptoms. Improving nutritional status and increasing GNRI scores may not only alleviate depression but also enhance overall patient health. Moreover, patients with low GNRI scores frequently exhibited higher rates of sarcopenia, highlighting a comorbid relationship between muscle loss and depression. Specifically, 83.90% of depressed patients also had sarcopenia, suggesting that reduced physical activity and inadequate nutrient intake in depression may promote muscle wasting, further exacerbating the vicious cycle between physical decline and depressive symptoms. Sarcopenia emerged as another significant predictor of depression. Patients with sarcopenia had notably higher depression scores than those without, indicating a strong link between muscle loss and depressive symptoms. Sarcopenia not only reflects deteriorating physical health but may also worsen depression by impairing quality of life and physical function (36, 37). In contrast, among patients with different stages of CKD, both depression and hopelessness were found to predict the occurrence of sarcopenia. Therefore, depression can act as a reverse predictor for sarcopenia in CKD patients. Providing antidepressant therapies or goal-oriented educational programs to alleviate depression or hopelessness can be effective strategies for preventing sarcopenia (5). Interestingly, although clinical and biochemical markers (e.g., creatinine, blood urea nitrogen, albumin) did not differ significantly, sarcopenic patients had lower GNRI scores, suggesting a combined effect of malnutrition and muscle loss on the development and severity of depression. This highlights the importance of early screening and comprehensive interventions targeting both sarcopenia and depressive symptoms to improve physiological and psychological outcomes.

Furthermore, after adjustment of other confounders, nutritional status and sarcopenia still exhibited independently and significantly associated with depressive symptoms in MHD patients. These findings highlight the complex relationship between poor nutritional status, sarcopenia, and depressive symptoms in elderly Chinese patients undergoing hemodialysis. Several potential pathophysiological mechanisms may explain how these factors contribute to mental health issues in this population: (1) Impact of nutrition on neurotransmitter function: Malnutrition, particularly protein-energy deficiencies, can impair the synthesis of neurotransmitters such as serotonin, dopamine, and norepinephrine, which are critical for mood regulation. In MHD patients, poor nutrition, compounded by the stress of dialysis, may disrupt brain chemistry, increasing the risk of depressive symptoms (38, 39). (2) Inflammation and oxidative stress: Both malnutrition and sarcopenia are linked to increased systemic inflammation and oxidative stress, which are known to contribute to depression. Inflammatory cytokines (such as TNF-*α* and IL-6) released during dialysis can interfere with neurotransmitter function, while oxidative stress can damage brain cells, leading to cognitive decline and depressive symptoms (40, 41). (3) Impaired immune function and neuroinflammation: Nutritional deficiencies can weaken the immune system, contributing to neuroinflammation in MHD patients. Chronic immune activation, coupled with poor nutrition, may enhance neuroinflammatory processes, affecting brain regions involved in mood regulation, and thus increasing the risk of depression (42). (4) Declining physical function, hormonal changes, and body composition: Sarcopenia, or the loss of muscle mass, can severely impair physical mobility, reducing patients’ ability to engage in daily activities and social interactions. This decline in physical function can lead to feelings of helplessness, which, combined with a reduction in muscle-derived neuroprotective myokines, can exacerbate depressive symptoms (43). Sarcopenia and malnutrition lead to changes in body composition, notably an increased fat-to-muscle ratio. These changes can disrupt hormone levels, including cortisol, leptin, and ghrelin, all of which play roles in mood regulation (18). Altered hormonal balance, along with the physical discomfort caused by muscle weakness, may contribute to depressive symptoms.

In addition, renal function markers, such as creatinine and blood urea nitrogen, were also closely associated with depressive symptoms. Declining kidney function may contribute to the onset of depression, particularly in patients with advanced renal impairment (44). After adjusting for renal function, correlations between depressive symptoms and other clinical parameters, such as albumin and hemoglobin, were no longer significant. This indicates that depression in MHD patients is influenced by a complex interplay among renal function, malnutrition, and sarcopenia rather than by traditional nutritional or laboratory indicators alone. Furthermore, it is noteworthy that in our adjusted models, some traditional nutritional biomarkers (e.g., hemoglobin, lipid profiles) were not independently associated with depressive symptoms or sarcopenia. This may be because GNRI, as a composite index incorporating both albumin and body weight relative to ideal weight, provides a more robust and holistic reflection of overall nutritional risk in the elderly, capturing aspects not fully represented by single biochemical parameters. Furthermore, the chronic inflammatory state prevalent in MHD patients, indicated here by CRP’s significant association, might confound or attenuate the direct relationship between isolated nutritional markers and psychological or musculoskeletal outcomes. The complexity of the malnutrition-inflammation-atherosclerosis (MIA) syndrome in ESRD can mean that the pathways linking nutrition to depression and sarcopenia are heavily mediated by inflammation, which our model partially accounts for through CRP. Given the close associations among malnutrition, sarcopenia, and depressive symptoms, early identification and intervention are essential. Tools such as the GNRI can help detect high-risk patients, allowing timely implementation of nutritional support and muscle-strengthening interventions. Regular monitoring of renal function, particularly creatinine levels, can also help identify patients susceptible to depression due to worsening kidney function. Multidisciplinary interventions addressing both nutritional deficits and sarcopenia can substantially improve the physical and mental well-being of MHD patients.

Overall, this study indicates that nutritional status, sarcopenia, and depressive symptoms are significantly interconnected among elderly Chinese MHD patients, which highlights the critical role of improving nutritional status and addressing sarcopenia in alleviating depressive symptoms in this population. Targeted interventions that enhance nutrient intake and promote muscle rehabilitation can significantly improve depressive symptoms and overall quality of life. Future research should further investigate the long-term effects of these interventions and refine treatment strategies to provide more precise care for this high-risk population.

## 5 Limitations

Despite the valuable insights provided by this study, several limitations should be acknowledged: (1) Cross-sectional design: This study’s cross-sectional nature limits our ability to establish causal relationships between nutritional status, sarcopenia, and depressive symptoms. Longitudinal studies are needed to determine the directionality and long-term impact of these factors on mental health in MHD patients. (2) Generalizability: The study was conducted in a specific cohort of elderly Chinese patients undergoing hemodialysis. Therefore, the findings may not be generalizable to other populations with different ethnicities, cultural backgrounds, or healthcare settings. (3) Self-reporting bias: Depressive symptoms were assessed based on patient self-reports, which might be subject to bias or underreporting, especially in a population with severely impaired cognitive function or social stigma associated with mental health. Future studies can benefit from using standardized diagnostic interviews for a more accurate assessment of depression. (4) Unmeasured confounders: While we adjusted for several clinical, biochemical, and demographic factors, there might still be unmeasured confounders, such as social support, medication use, or other psychological conditions, that could influence the observed associations between nutritional status, sarcopenia, and depression. (5) Although the scales used in this study were standardized instruments, their reliability (such as Cronbach’s *α*) was not re-evaluated in our specific sample, which represents a limitation.

## 6 Conclusion

This study demonstrates that both poor nutritional status and sarcopenia are independently and significantly associated with depressive symptoms in elderly Chinese patients undergoing hemodialysis. These findings indicated that lower GNRI scores, which reflected poorer nutritional status, were strongly associated with higher odds of depression, particularly in patients in the lowest GNRI quartile. Furthermore, sarcopenia, characterized by muscle loss and reduced physical function, was also found to be a major contributor to depressive symptoms in this population. The robust associations observed between nutritional status, sarcopenia, and depression, even after adjusting for various clinical and biochemical factors, highlight the complex interplay between these factors in influencing mental health outcomes in MHD patients. Given the high prevalence of both malnutrition and sarcopenia in this population, addressing these issues through targeted nutritional interventions and rehabilitation programs may be crucial for improving the overall well-being and mental health of MHD patients. Moreover, these findings underscore the need for comprehensive management strategies that go beyond dialysis and kidney function alone, incorporating nutritional support and muscle preservation efforts to mitigate the psychological burden on these patients. Future longitudinal studies are warranted to explore the causality of these associations and evaluate the effectiveness of interventions aimed at improving nutritional status and reducing sarcopenia in preventing or alleviating depressive symptoms in MHD patients.

## Data Availability

The raw data supporting the conclusions of this article will be made available by the authors, without undue reservation.
