# Correction: The association between nutritional status, sarcopenia, and depressive symptoms in elderly Chinese patients receiving maintenance hemodialysis: a hospital-based cross-sectional study

**DOI:** 10.3389/fnut.2026.1819107

**Published:** 2026-03-16

**Authors:** Lin Huang, Yan Zhang, Jinbao Wang, Jiajun Zhou

**Affiliations:** 1 Blood Purification Center, The First Affiliated Hospital of Wannan Medical College (Yijishan Hospital of Wannan Medical College) Wuhu, China; 2 Department of Nephrology, The Second Affiliated Hospital of Anhui Medical University, Hefei, China

**Keywords:** association, depressive symptoms, maintenance hemodialysis, nutritional status, sarcopenia

Affiliation Blood Purification Center, The First Affiliated Hospital of Wannan Medical College (Yijishan Hospital of Wannan Medical College) Wuhu, China was erroneously given as Blood Purification Center Affiliated Yijishan Hospital of Wannan Medical College, Wuhu, China for all authors.

The original version of this article has been updated.

